# Particle Therapy for Breast Cancer: Benefits and Challenges

**DOI:** 10.3389/fonc.2021.662826

**Published:** 2021-05-05

**Authors:** Wanrong Luo, Yasser F. Ali, Chong Liu, Yuchen Wang, Caorui Liu, Xiaoni Jin, Guangming Zhou, Ning-Ang Liu

**Affiliations:** ^1^ State Key Laboratory of Radiation Medicine and Protection, School of Radiation Medicine and Protection, Collaborative Innovation Center of Radiological Medicine of Jiangsu Higher Education Institutions, Soochow University, Suzhou, China; ^2^ Biophysics Lab, Physics Department, Faculty of Science, Al-Azhar University, Cairo, Egypt

**Keywords:** breast cancer, carbon ion radiotherapy, proton therapy, particle therapy, particle treatment-associated challenges

## Abstract

Hadron therapy with protons and carbon ions is widely attracting interest as a potential competitor of conventional photon radiotherapy. Exquisite dose distribution of charged particles allows for a higher local control of the tumor and lower probability of damage to nearby healthy tissues. Heavy ions have presumed biological advantages rising from their high-linear energy transfer (LET) characteristics, including greater cell-killing effectiveness and reduced heterogeneity dependence of radiation response. Although these advantages are clear and supported by data, only 18.0% of proton and carbon ion radiotherapy (CIRT) facilities in Europe are treating breast cancers. This review summarizes the physical and radiobiological properties of charged particles, clinical use of particle beam for breast cancer, and suggested approaches to overcome technical and financial challenges.

## Introduction

Breast cancer, ranking first in incidence and mortality, threatens women’s health globally. In the year of 2020 alone, 2.2 million female breast cancer cases were newly diagnosed (1). Surgery represents the mainstay of curative therapy for breast cancer. However, the delivery of adjuvant radiation following lumpectomy has been shown to improve survival and decrease recurrence. A meta-analysis of 10801 breast cancer patients showed that radiotherapy after mass resection can effectively reduce the 10-year recurrence rate and 15-year mortality rate (2). Interest in new radiotherapeutic techniques that minimize deposited doses in proximal normal structures as targets, with the goal of reducing acute and late adverse effects of treatment, has risen in recent years. Particle therapy with protons and heavy ions meets such criteria. The dose deposition of carbon-ion beam, for example, is low in the entrance region where the normal tissue is exposed, but greatly enhanced at the end of their range where the tumor is located, and drops to near zero abruptly thereafter (3). This greater ionizing density within the targeted neoplasm induces a higher degree of irreversible DNA lesions, which overwhelms cancer cells’ repair capacity. The relative biologic effectiveness (RBE) concept has been introduced to account for this increased efficiency of cell killing. At clinical institutions, an RBE value of 1.1 to 1.2 has been documented for proton therapy, while for the heavier carbon ions, the RBE distribution in the targeted tissues varies between 2 and 5 (4). The direct-killing effect of heavy ions maintains their ability to kill malignant cells irrespective of oxygen concentration. Therefore, even hypoxic tumor tissues are significantly sensitive to carbon ion therapy (5).

Historically, particle facilities are rarely used in the treatment of breast cancer. Just two out of the 11 European centers were treating breast cancer patients. Charged particles has a physical and biological advantage that can be utilized clinically in treating a substantial number of patients with breast cancers in the future.

## Clinical Outcomes for Proton Therapy

With the rapid introduction and widespread usage of particle therapy, epidemiologically frequent tumors have been explored. The number of patients treated with proton therapy per year increased from 16,200 in 2015 to approximately 190,000 in 2018 and is expected to grow to be over 300,000 by 2030 (6). In 2014, Bush et al. (7) reported the outcomes of proton therapy (PT) on 100 patients diagnosed with invasive non-lobular carcinoma and a maximal tumor dimension of 3 cm. Postoperative proton beam was given at a dose of 40 Gy in 10 fractions, once daily over 2 weeks. Following treatment, patients underwent a 5-year follow-up period to monitor toxicity and tumor recurrence. Proton treatment produced excellent ipsilateral breast recurrence-free survival of 97% and overall survival of 95% with minimal toxicity. There were no cases of grade 3 or higher acute skin reactions. Furthermore, comparative study has been conducted to estimate the gain obtained with PT in term of recurrence and cardiotoxicity risk using clinical data of EORTC 22922/10925, and NCIC-CTG MA.20, including 41 patients of locally advanced breast cancer with nodal involvement. The median estimated excess of absolute risk of breast cancer recurrence after 10 years was 0.10% with photons versus 0.02% with protons (8).

Heart and lungs lie directly underneath the breasts and are likely to receive some radiation too. Total lung doses exceeding 10 Gy has been reported in patients with unilateral mammary carcinoma who received adjuvant RT (9). Women irradiated for left-sided breast cancer receive substantially higher doses to the heart compared to those who irradiated for right-sided breast cancer. Systematic review documented that the average cardiac dose was 5.4 Gy for left-sided breast irradiation compared to a dose of 3.3 Gy in case of right-sided irradiation. Hooning et al. (10), have found an increased development of myocardial infarction and congestive heart failure due to incidental cardiac radiation exposure during internal mammary chain (IMC) treatment. Strikingly, the proton beam significantly lowers mean doses to lung and heart compared to even the most optimized photon beam plan, data are summarized at **Table 1**, and enables patients to undergo a limited risk of cardiac toxicity (16). Coronary arteries as well are at risk in radiation therapy of breast cancer. A study of 199 patients with breast cancer detected a four to seven-fold increase in incidence of stenosis in mid and distal left anterior descending artery (LAD) artery after RT of left-sided compared to right-sided breast cancer (17).

**Table 1:** Overview of dose-volume histograms for breast target coverage (PTV) and adjacent critical organs in the proton-photon planning comparison literature.

Study	PTV			OARs					
	Photon	IMRT	Proton	Mean Heart dose			Mean dose to Ipsilateral Lung		
				Photon	IMRT	Proton	Photon	IMRT	Proton
Fogliata et al. (11)	V_95%_ = 96.3	V_95%_ = 94.7	V_95%_ = 99.8	2.7 Gy	2.8 Gy	2.2 Gy	12.9 Gy	9.2 Gy	3.5 Gy
Johansson et al. (12)	V_95%_ = 93.2	V_95%_ = 85.9	V_95%_ = 94	30.5 Gy	20.5 Gy	10.5 Gy	14.5 Gy	9 Gy	0.5 Gy
Lomax et al. (13)	V_95%_ = 86.6	V_95%_ = 92.2	V_95%_ = 97.1	15 Gy	16 Gy	6 Gy	17 Gy	15 Gy	13 Gy
Ares et al.* (14)	V_95%_ = 95	V_95%_ = 99	V_95%_ = 100	9 Gy	12 Gy	1 Gy	17 Gy	15 Gy	7 Gy
Sun et al.* (15)		V_100%_ = 96.9	V_100%_ = 97.6		498.4 cGy	94.6 cGy		881.8 cGy	414.8 cGy

PTV, planning target volume; OAR, organ at risk; IMRT, intensity-modulated radiotherapy; V_95%_ V_100%_, volume that receives at least 95% and 100% of the prescribed dose respectively.

*reports on sophisticated mode proton beam of variable energy and intensity, IMPT, intensity-modulated proton therapy.

Patients may be at risk of locoregional recurrence (LRR) after mastectomy due to microscopic residual disease areas in the chest wall and draining lymphatics. Post-mastectomy radiotherapy (PMRT) has been found to reduce LRR risk and potentially improve survival (18). Of note, Protons-PMRT provides excellent locoregional control rates and nearly complete avoidance of cardiopulmonary structures compared to photon-based approaches (19). Additionally, proton fields are delivered en face, allowing for an arms down position (20). Breast reconstruction following a mastectomy has become an integral component of the treatment process for breast cancer. Immediate breast reconstruction undoubtedly maintains body image as well as quality of life, which remains relatively unperturbed, but the opportunities of such an approach must be weighed against potential oncologic and toxicity challenges (21). Smith and colleagues similarly reviewed outcomes of 51 patients who underwent immediate implant reconstruction with adjuvant proton therapy (22). Reconstruction failure occurred in 15% of patients, raising concerns regarding toxicity with this approach. Thus, patients are often recommended to delay reconstruction.

Re-irradiation (reRT) may be an optimal treatment for recurrent breast cancer, but is challenged by fear of excessive toxicities and inability to safely deliver definitive (≥60 Gy) doses of reRT (21). Based on the favorable physical property of the Bragg peak, proton beam therapy is particularly well suited for reirradiation that has indeed been reported in multiple disease sites, including central nervous system, head and neck, gastrointestinal, and lung tumors, reviewed at (23). A study of 92 patients with recurrent head and neck cancer showed that reRT with PBT is safe and effective. The treatment was deemed effective since Locoregional failure at 1 year was only 25%, combined with a low risk of late grade 3 or 4 dermatitis and dysphagia (8.7% and 7.1%, respectively) (24). Notably, limited data exist for the use of PBT in the setting of reRT for breast cancer. A multi-institutional report of 50 patients who underwent breast cancer proton re-irradiation showed a favorable local control at a median follow-up of 12.7 months. Of note, the 1-year local recurrence free survival (LFRS) and overall survival (OS) were 93% and 97%, respectively, with only 16% of patients experiencing grade 3 adverse events (25). A recent retrospective analysis of 74 patients treated with PBT for reRT showed a similarly low rate of grade 3 toxicity at any point (13%) (26), which compared favorably with historical toxicity incidences for photon re-irradiation (27).

## Clinical Outcomes for Heavy Ion Therapy and Neutron Radiation

The experience of using carbon ion therapy for breast cancer has been limited, mostly due to the restricted number of qualified facilities and the cost burden of treatment. Nine cases out of a total 11,580 cancer patients were treated with carbon ions at the National Institute of Radiological Sciences (NIRS) between June 1994 and July 2017 in Japan (28). The first published case report of the effectiveness of carbon ions for the treatment of stage I breast cancer without surgery at NIRS was in 2014. Medical examination revealed that the patient had T1N0M0 estrogen receptor-positive invasive ductal carcinomas (24). As a follow-up on this report, Karasawa et al. (29) designed dose escalation study on seven patients with stage I breast cancer, with dose levels of 48.0, 52.8 or 60.0 Gy (RBE), administered in four fractions within 1 week. Three months after radiotherapy, the patients underwent tumor excision for pathological evaluation. Eligibility criteria were being a woman of age 60 years and over, had a life expectancy of over 6 months, had a solitary tumor with a diameter of under 2 cm, and being estrogen receptor (ER) positive and human epidermal growth factor receptor 2 (HER2) negative. Three patients received 48 Gy (RBE), three received 52.8 Gy (RBE), and one received 60.0 Gy (RBE). Following irradiation, four patients experienced acute grade 1 skin toxicity, but no other toxicities reported. At 3 months after the carbon ion radiotherapy, most patients had a pathologic response to treatment. At the follow-up of 37–48 months, all patients were alive with no clinical evidence of recurrence or subsequent effects. In a recent analysis of 14 patients who underwent C-ion RT for stage I breast cancer, complete tumor disappearance occurred for longer than expected, 2 years after treatment, with only mild adverse events experienced by 10 patients. One recurrence case was noted at 6 months after treatment and the patient eventually died of systematic metastases, while others survived without recurrence and had good cosmetic outcomes at a median follow-up of 5 years (30). Compared to treatment results obtained with radiofrequency ablation (RFA), cryoablation therapy, high-intensity focused ultrasound (HIFU), and stereotactic body radiotherapy (SBRT), C-ion RT is considered to have merits of high tumor control and low adverse events. Harada et al. (31) similarly reported on patients with breast cancer who underwent CIRT at a single dose of 36 Gy. Patients tolerated treatment well and survived more than 8 years without local recurrence, providing additional support of carbon-ion radiotherapy’s usefulness and safety.

Besides CIRT, neutron therapy is another appropriate clinical choice for the tumors radioresistant to the conventional photon therapy. Neutron therapy is a type of radiation treatment by using a stream of high-LET subatomic particles with no electric charge, to target and destroy tumor cells. Initial experience with the neutron beam showed a benefit in terms of local control in breast tumor. A prospective randomized study included 29 patients with locally advanced breast cancer (T4, NO-3, MO-1), compared to a dose level of 17 with 19 Gy for local control and morbidity. Neutron treatment gave a local control rate of 68% for the 17 Gy and 83% for the 19 Gy arm. Following irradiation, few cases of grade 4 skin and subcutaneous necrosis were observed in the 19 Gy arm and none in the 17 Gy (32). A randomly controlled trial on locally advanced breast cancer compared neutron with conventional photon irradiation in terms of tumor control and late radiation morbidity. Although no significant differences were observed between the two treatment groups, neutron therapy improved the quality of life due to its shorter treatment duration (33).

## Biological Advantages of Charged Particles in Breast Cancer Treatment

Evidence indicates that breast cancer is composed of phenotypically diverse groups of neoplastic cells, within which there is a small subpopulation termed cancer stem cells (CSCs). CSCs are thought to have the characteristics of self-renewal and therefore play a pivotal role in tumor development, progression, and recurrence after treatment (34, 35). Breast cancer cells that have a high expression of CD44 together with low level of CD24 have been reported to have stem cell properties and to have a higher tumorigenic capacity than other cells (36). Clinically, CD44/CD24 expressions are widely used as CSC markers in breast cancer. Around 40% of all breast malignancy and half of the locally advanced breast cancers contain regions of intratumoral hypoxia with an oxygen concentration below that of normal mammary tissue (37, 38), perhaps through promoting CSC maintenance (39) that consequently makes tumors radio- and chemo-resistant. A preliminary study indicates an increased effectiveness of low-energy protons in eliminating CSCs from mammary human cancer cells *in vitro* (40). Intriguingly, it showed a greater capability of eliminating CSCs than photon-based therapy at the same dose (41), therefore, it has substantial clinical advantages on mammary tumors with hypoxic fractions. Such better performance is explained by differences in induction and repair of DNA damage. Held et al., observed more unrepaired clustered DNA lesions after carbon-ion irradiation, while almost all sporadic DNA damage sites were efficiently rejoined after the same dose of X-rays treatment (42).

Increasing evidence reveals that mutations in the tumor suppressor p53 gene are the most common, occurring in over 50% of human cancers. Tumors with p53 mutation(s) are more resistant to radiotherapy by abolishing the p53 dependent apoptosis in response to radiation exposure (43–45). In breast cancers, p53 is mutated in almost 30% of clinical cases and found associated with an elevated risk of mortality (2.27-fold) compared with patients with no such mutation (46, 47). Compared with conventional X-ray treatment, carbon ions showed the advantage of overcoming radioresistance; it effectively induces p53-independent apoptosis (48) *via* E2F1 signal pathway (49). Upregulation of E2F1 protein expression caused a higher reduction in clonogenic survival, G2/M phase arrest, promotion of apoptosis rate, up-regulation of phosphor-Rb, Bax, and cleaved-caspase3 proteins expressions without p53.

## Combination Strategies to Improve Charged Particle Treatment Efficacy

Even though local control is generally very high with particle therapy, combination of systemic therapies and irradiation become the recommended treatment option for most malignancies to control metastasis and increase survival (50). In vitro experiments on breast cancer cells provided useful indications on the combination of different antineoplastic agents with CIRT. Combination therapy resulted in greater cytotoxicity toward human mammary epithelial cancer line MCF7 and showed promise in facilitating the delivery of antitumor drugs (51). Few preclinical studies have been published to investigate the underlying mechanism and efficacy of carbon ion radiation in combination with immunotherapy (IT) (52). Carbon-ion irradiation resulted in tumor elimination, rejection of secondary tumor inoculation, and T-cell activation. These antitumor effects were enhanced by the combination of dendritic cells (DCs) as IT. Indeed, more preclinical research containing large-sized samples, diverse immunotherapies, various radiation doses, and fractionations needs to be done before CIRT achieves its promise.

Another treatment combination is CIRT and chemotherapy which has shown great outcomes in preclinical studies for cancer cures (53). Combined protocol efficiently kills triple-negative breast cancer (TNBC) stem cells *in vitro*, likely due to suppressed colony formation, inhibited cell cycles, irreparable DNA lesions, and enhanced apoptosis compared to X-ray combined with cisplatin or carbon ion beam alone.

Co-treatment with PU-H7, a Hsp90 Inhibitor, sensitizes human cancer cells to carbon-ion irradiation by inhibiting homologous recombination (HR) and non-homologous end joining (NHEJ) DSB repair pathways. While the radiosensitization effect of PU-H71 was not observed in human normal cell lines (54). This selective effect illustrated an impressive and encouraging outcome for future protocols combining particle therapy and Hsp90 inhibitors.

## Current Issues With The Use of Particle Therapy

Breast cancer poses a challenging problem in accurate dose calculation due to respiratory-induced motion. Motion-induced uncertainty compromises CT geometrical information of the target and nearby normal organs as the CT reconstruction algorithm was developed for static objects (6). Respiratory Gating and Breath hold techniques including breath-hold-at-inhalation (BHI), and breath-hold-at-exhalation (BHE) are various anatomic motion management strategies, currently in use to mitigate the effects of thr breathing motion. A clinical practice shows that significantly low doses to organs at risk are observed in an enhanced end-inspiration phase and the risk of cardiopulmonary complications was 1% or less (55). In contrast, Breast radiotherapy with scanned proton beams results in better dose coverage of regional lymph node targets and lower doses delivered to critical structures irrespective of whether respiratory gating is used or not (56). Indeed, Proton spot scanning seems to maintain a low cardiovascular burden beyond what could be achieved with enhanced inspiration gating and photon therapy (57).

Cost is the main barrier for the diffusion of particle therapy service; most of the expense being for the construction of accelerating structure and rotating gantries to focus the beam down to the patient. Proton gantry size is still a limiting factor; most gantries have diameters of several meters, owing to magnet coils required to bend the beam path. However, Superconducting magnets reduced gantry size and weight to practical proportions and decreased the demands on the mechanical structure (58). Further technological efforts based on robotic applications are made up in order to resolve cost barriers. Briefly, fixed-beam treatment rooms, where the patient is rotated and translated in space with a robotic arm solution to enable beam incidence from various angles for optimal target coverage (59). Nevertheless, gantry replacement by one or a few fixed beams has been argued to result in sub-optimal treatments in a significant proportion of cases (60) but this depends on the kind of technology adopted for positioning.

## Conclusion

The ballistic and radiobiological properties of particle beam make it a potential treatment option for radioresistant breast cancer subtypes. it could be more cost efficient than photons in patients who are at risk of cardiovascular disease. Further development on motion mitigation strategies and tracking algorithms are required to minimize range uncertainties that ultimately help to translate the dosimetric advantages to clinical benefit.

## Author Contributions

WL and YA drafted the paper. N-AL and GZ developed the final version. CL, YW, CRL and XJ contributed to writing and to the critical review of the manuscript. All authors contributed to the article and approved the submitted version.

## Funding

This work was supported by the Suzhou Science and Technology Development Project (SYS2020090), the Program of the Network-type Joint Usage/Research Center for Radiation Disaster Medical Science, the Collaborative Innovation Center of Radiological Medicine of Jiangsu Higher Education Institutions, and a project funded by the Priority Academic Program Development of Jiangsu Higher Education Institutions (PAPD).

## Conflict of Interest

The authors declare that the research was conducted in the absence of any commercial or financial relationships that could be construed as a potential conflict of interest.
